# Evolution of attack in handball when playing 7 vs. 6 with empty goal between 2020 and 2023: coaches’ perception vs. observational results

**DOI:** 10.3389/fspor.2024.1354623

**Published:** 2024-03-14

**Authors:** João Nunes Prudente, Américo Ramos Cardoso, Ana José Rodrigues, João N. Mendes, Catarina Fernando, Helder Lopes, Alejandro Trejo-Silva, Duarte Filipe Sousa

**Affiliations:** ^1^ Department of Physical Education and Sport, Faculty of Social Sciences, University of Madeira, Funchal, Portugal; ^2^ Centre for Tourism Research, Development and Innovation, University of Madeira, Funchal, Portugal; ^3^ Higher School of Technologies and Management, University of Madeira, Funchal, Portugal; ^4^ Research Center in Sports Sciences and Human Development, University of Trás-os-Montes and Alto Douro, Vila Real, Portugal; ^5^ Instituto Superior de Educación Física, University of the Republic, Montevideo, Uruguay; ^6^ Young Men's Christian Association University Institute, Montevideo, Uruguay

**Keywords:** men’s handball, mixed-methods, observational methodology, polar coordinate, questionnaire, attack with empty goal, coaches’ perception

## Abstract

**Introduction:**

Recently, several studies on the 7 vs. 6 “empty goal” (EG) in handball have produced different and even contradictory results. The aim of the present study was to investigate the behavior of teams and players in the 7 vs. 6 EG attack in the European (Euro) and World Championships (WCh) between 2020 and 2023 and characterize the coaches’ perceptions.

**Methods:**

A mixed-methods approach was used, consisting of the following: (i) an observational methodology and instrument developed and validated to collect observational data on player and team behavior; and (ii) a developed and validated questionnaire to coaches on their perceptions of the 7 vs. 6 game. Observational data were collected during the Euro 2020 and 2022 games (*n* = 62) and the WCh 2021 and 2023 games (*n* = 70). A total of 132 games and 391 situations of 7 vs. 6 attacking sequences were observed. In total, 156 coaches participated (146 men), with a mean age 42.33 ± 11.87 years, 19 nationalities, and with 12.77 ± 9.45 years of experience.

**Results and discussion:**

The choice of 7 vs. 6 offensive play was mostly made in the second half (>73%). The effectiveness of 7 vs. 6 offensive sequences was higher in the top six teams than in the team's ranked 7th to 12th (Euro 2020 51.6%–50.0%; WCh 2021 52.0%–50.0%; Euro 2022 53.1%–41.7%; WCh 2023 50.0%–43.8%). Some patterns of association were found (*p* < 0.05 and with values >±1.96): (i) scoring a goal with a breakthrough shot was significantly associated with the effectiveness of the 7 vs. 6 attack (Euro 2020 2.61; WCh 2021 2.87; Euro 2022 2.68; WCh 2023 2.32); (ii) teams in the top six significantly used 7 vs. 6 when they were winning (Euro 2020 2.17; WCh 2021 3.52; Euro 2022 5.88; WCh 2023 2.54); and (iii) teams in the bottom six used it when they were losing by at least four goals (Euro 2020 7.56; Euro 2022 6.64; WCh 2023 4.37) or when they were winning by four goals or more (WCh 2021 2.58). Coaches that agree with the possibility of playing 7 vs. 6 (74.4%), rarely or never do so (55.6%) because it brings little or no advantage (52.6%). The results of the analysis confirmed the perception of the coaches, the low use of 7 vs. 6, the low advantage associated with it, and the influence of the result and the moment of the game on its use.

## 1 Introduction

Team sports, such as handball, involve complexity, opposition, and cooperation. They are characterized as being interactive, with players interacting with each other, both teammates and opponents, and with context, whether at the location of the game, in the area of the field where the action takes place, in the elapsed game time, in the partial result, or in the numerical relationship (1–3). Players’ behaviors are generally emergent, deriving from individual characteristics, as well as from the possibilities that the context offers and from the characteristics of the tasks performed by the players (4–7). Handball is a complex system, as defined by Balagué et al. (8), in which strategic tactical behavior is crucial (9–12) and performance is the result of interaction between different factors and variables, including the numerical relationship. Currently, handball games with different numerical relations are frequent, due to the characteristics of the rules of the game—with their progressive penalties, and the possibility of players being punished with 2-min exclusions, with disqualification due to accumulation of exclusions and with direct disqualification (3). These authors stated that, having different symmetrical and asymmetrical numerical relationship in the number of players, is clearly a characteristic of the current handball game, which has been increased in recent competitions (13, 14), especially after the changes of the rules in 2016, with the possibility of playing in attack with an empty goal (EG) (13). The most common relationships are still 6 vs. 6, 6 vs. 5, 5 vs. 6, and 5 vs. 5 with goalkeepers at goal, but since the change of the goalkeeper rule, the 7 vs. 6 and 6 vs. 6 game situations with an EG are becoming more common and have attracted the attention of researchers with some published studies, with 7 vs. 6 being the situation that has focused the most attention. In the last 7 years, several studies have been carried out on the 7 vs. 6 with an “empty goal” (7 vs. 6 EG) game. The results obtained, varied and even contradictory among them, are in line with the controversy that arose when the regulatory change allowing 7 vs. 6 situations was approved (7, 15–24). Regarding 7 vs. 6 EG, it is important to note that the majority of some studies found and analyzed observational procedures; regarding 7 vs. 6 EG, it is important to note that most of the studies found follow observational procedures. Only a few studies analysed the opinion and perception of the coaches (22, 23, 25–29) and only one study was found that considers the opinion of the players (24). As observed by Korte and Lames (30), with this new rule of attacking with an “empty goal,” different attacking formations have increasingly occurred, such as seven attacking players (with two pivots or with one pivot) vs. six defenders. This reality was complemented by Maroja et al. (18), who observed that losing teams tend to use this strategy more than winning teams (9.7% and 3.9%, respectively) during the play-off stage at the 2017 Women's Handball World Championship (WCh). The use of the rule to attack with seven players and an empty goal generated controversy within elite handball, discussing the risk assumed by the teams that opt for this tactical option (25, 26), in concordance with Antón (31). In the research carried out by Bonjour et al. (32), with a sample made up of 571 attack sequences with an empty goal, referring to 50 games of the 2018–2019 European Handball Federation (EHF) Women's Champions League, the results showed that the teams managed to significantly recover defensively, in an adequate manner, and organize their defense. However, they recorded a considerable number of direct goal-to-goal shots against (15.7%), with an effectiveness level of 57%. The authors concluded that teams, when attacking with an EG, take on a significant risk of conceding a goal quickly if they do not score a goal. However, Gümüş and Gencoglu (17), when studying the effects of the goalkeeper substitution rule as a new strategy in handball, concluded that the teams that used this strategy, despite not having greater efficiency in attack, did not have negative consequences nor did they have an increase in risk when playing 7 vs. 6 EG. Krahenbühl et al. (25, 26) pointed out these main conclusions: coaches considered there were no significant strategic changes in handball neither in attack nor in defense, and that the additional court player was used to maintain the numerical equality in the attack in situations of exclusion and, in some specific cases, aimed at numerical superiority in final and decisive moments of matches. Prudente et al. (27) studied the coaches’ perception on playing 7 vs. 6 EG and stated that the majority (65.8%) of Portuguese coaches considers that game time influences the use of the “7 vs. 6 EG” strategy, and 92.2% of them stated that this use occurs in the final moments of the game. Coaches (74.7%) have the perception that the result influences the use of 7 vs. 6 EG, with 90.7% considering that being behind in the score positively influences its use. Sousa et al. (23), in their study about the Portuguese coaches’ perception about play 7 vs. 6 EG, concludes that most coaches (86.3%) agree with the possibility of using this strategy. Most respondents (70.9%) do not think that the game is mischaracterized, but only 13% always or often use this possibility and three-quarters do not agree with the elimination of the rule. Antón (33) stated that the rule is strategically used as often as in every game of a national team's tournament, such as the Portugal Men's National Team at the 2020 Men's EHF Euro, where Portugal even played a complete match using this tactic. To deepen our knowledge of the topic and its implications for the current handball game, we decided to study the behaviors of teams and players, in a 7 vs. 6 situation with an empty goal, over the last 4 years of high-level international competitions, at the Men's European Championship (Euro) and Men's WCh, more precisely between 2020 and 2023, crossing the observation results with the coaches’ perceptions about playing 7 vs. 6 EG.

## 2 Material and methods

The study uses a double methodology: (i) observational methodology, using an idiographic (I)/multidimensional (M)/follow-up (F) observational design; idiographic due to the fact that all the sequences 7 vs. 6 were observed as a unit in the same competition; multidimensional (M) in that several response levels were studied; and follow-up (F) as several games played in the same championship were observed; having then data located in the first quadrant and type 1 data (34, 35). Data from games were collected using an *ad hoc* observation instrument built and validated for this purpose; (ii) the application of a questionnaire on the coaches’ perception of this new rule and its influence on the game, which was constructed and validated using the Delphi method. Regarding the observational instrument, the one used by Prudente et al. (7), in their study of the use of 7 vs. 6 EG during the 2020 EHF Men's Euro, was applied to collect data from games. Moreover, coaches’ opinion and perception were registered via a questionnaire compiled using Google Forms. Given its characteristics, high scientific rigor, flexibility, and the allowance of an objective study of spontaneous behavior in natural settings, the observational methodology has become one of the preferred methods in scientific research in sport and team games, particularly in handball, which has been using it in recent decades (36, 37). Mixed-methods studies are being increasingly applied to a diversity of fields and have enormous potential in the field of sport and physical activity (37, 38). In the present study, a mixed-methods approach was used. Through systematic observation, data from the behavior of players and teams in a competitive environment were registered. With the qualitative data obtained, a quantitative analysis was then carried out, using the lag sequential analysis and polar coordinate analysis techniques. Through a survey of coaches, data on their perception regarding the game 7 vs. 6 EG were obtained, allowing the cross-referencing of information obtained with the observational data, completing the information and interpretation of the data.

### 2.1 Sample

#### 2.1.1 Observational sample

The observational sample consisted of the total number of offensive sequences carried out while playing 7 vs. 6 EG, in the positional attack (*n* = 391), during the 132 games played by teams ranked 1–12 at 2020–2022 EHF Men's Euro (*n* = 62) and 2021–2023 IHF Men's World Championship (*n* = 70). All the sequences registered occurred in numerical superiority 7 vs. 6 with the team in possession of the ball playing without a goalkeeper at goal (EG), starting and ending with the previous numerical asymmetry mentioned. Table 1 presents the distributions of the offensive sequences in the four tournaments.

**Table 1:** Observational sample.

Competition	Euro 2020	WCh 2021	Euro 2022	WCh 2023
Games observed	34	34	28	36
No. of sequences 7 vs. 6 occurred	123	79	121	68

Euro, Men's European Championship; WCh, Men's World Championship.

#### 2.1.2 Instrument

The instrument used to collect data was a mixed *ad hoc* instrument consisting of a field format with category systems (7), with 11 criteria and 77 categories: (1) teams, according to final ranking at the competition (T1, T2, T3, T4, T5, T6, T7, T8, T9, T10, T11, T12); (2) game time, 10-min parts of the first and second halves until 50 min (A1, A2, A3, B1, B2), 5-min parts of the last 10 min of the second half (B3, B4) plus extra time (P1, P2, P3, P4); (3) the partial score (E, V1, V2, V3, V4, D1, D2, D3, D4); (4) defensive system (6:0, 5:1, 3:2:1, 3:3, 4:2, 5 + 1, 4 + 2, HxH); (5) offensive organization, with one or two pivots (1Pv, 2Pv); (6) tactical means, individual means, and group means (Ind, Group); (7) shot, from 9 m (RIL), wing shot (RPt), pivot shot (RPv), breakthrough shot (RPn), no shot (SR); (8) attack result: goal (G), no goal (NG), 7 meters with goal, (7MG), 7 meters no goal (7MNG), no shot by technical fault (SFFt), no shot by opponent action (SFAa); (9) the shot zone of the offensive sequence—a field map was designed with 10 zones: 9 zones on the offensive side (left/right wing, left/central/right zones from 6 to 9 m; left/central/right zones from 9 to 15 m; left/central/right zones from 15 to 20 m) and 1 zone on the defensive side; (10) opponent response: goal to goal attempt (GD), direct fast break (CAD), sustained fast break (CAA), throw-off (Rep), fast attack (AR), organized attack (AO), no response (NE); and (11) opponent response result: goal (Golo), no goal (Ngolo), no goal with penalty (NGcP), no action (SA).

#### 2.1.3 Procedures

Data were observed and recorded using Lince software v.1.2.1 (39). SDIS-GSEQ v. 5.1.23 (40) and Hoisan v. 1.6.3.3.5 software (41, 42) were used to analyze the data. The games were watched from recordings obtained from TV Broadcast and directly recorded on the MacBook Air 13" computer, 2017. Data quality control is a basic requirement in observational methodology (43); therefore, data reliability and observer reliability were corroborated using Cohen's Kappa (44), via the “Compute simple statistic” and “compute Kappa” functions of the GDEQ-SDIS v. 5.1.23 (40), assessing intra- and inter-observer agreement (Table 2). Two observers carried out all the observations and data recording, having tested intra- and inter-observer reliability before the start of the process. Before starting the observations, the two observers underwent a period of training to achieve observers’ reliability as recommended by Anguera et al. (45). After the training period, the observers carried out the observations and tested intra- and inter-observer reliability. Both observers obtained values above 0.79 in the Kappa test and held a session to discuss the recorded data and correct the recording criteria. An inter-observer Kappa test was also carried out and the value obtained (0.87) confirms inter-observer reliability. After they tested intra- and inter-observer reliability, they started to observe and register the games that were previously defined for each observer. Each observer was responsible for observing half of the games in the observational sample. During the process at the end of every four games (Euro 2020) and after every six games (Euro 2022, 2021, and 2023 WCh) observed, an intra- and inter-observer Kappa test was carried out. For the intra-observer test, the last game observed was used, and the observation was repeated 3 days later. For the inter-observer Kappa test, they previously defined which game would be observed by both observers to test inter-observer reliability. For intra-observer reliability, the minimum Kappa values were in the range of 0.79–0.91 and the maximum values were in the range of 0.91–0.97. For inter-observer reliability, the minimum values obtained between observers were in the range of 0.80–0.88, while the maximum values were in the range of 0.93–0.96. The above results for intra- and inter-observer reliability determine the quality of the data collected (46).

**Table 2:** Kappa values obtained (minimum and maximum).

Value of Kappa	Euro 2020	WCh 2021	Euro 2022	WCh 2023
Intra-observer	0.79–0.91	0.84–0.96	0.87–0.96	0.92–0.97
Inter-observer	0.87–0.94	0.80–0.94	0.80–0.96	0.88–0.93

Euro, Men's European Championship; WCh, Men's World Championship.

#### 2.1.4 Questionnaire

The questionnaire was constructed and validated using the Delphi method, by debate and consensus between five experts in the field of Sports Sciences and Handball. After designing the instrument, it was applied to a group of adult participants. This version of the questionnaire was subject to a pilot study. In the pilot study, the questionnaire was handed to 14 handball coaches, at two different times, with an interval of 7 days. This pilot study aimed to test the instrument's reliability, internal consistency, and the protocol procedures that support its application. There were high levels of reliability (intraclass correlation coefficient 1.000–0.902; *p* < 0.05) and internal consistency (*α* = 0.787, *p* < 0.05). The final version of the questionnaire, in Portuguese, English, French, and Spanish, consisted of 42 questions, with answers associated with a 5-point Likert scale (1 totally disagree to 5 totally agree), distributed across four sections: (i) personal data, training, experience, and current professional situation; (ii) perception and importance of 7 vs. 6 EG; (iii) the use of 7 vs. 6 EG in training and competition; and (iv) 7 vs. 6 EG in system offensive and defensive action.

#### 2.1.5 Questionnaire sample

The questionnaire was completed by 156 coaches of both sexes (146 men and 10 women) from three continents (Africa, Europe, and South America) and 19 nationalities, with a mean age of 42.33 ± 11.87 years. Most of the coaches had at least a level 3 (61.4%), a mean of 12.77 ± 9.45 years of experience, and had coached teams at the national (45.5%) or international (33.3%) level.

#### 2.1.6 Procedures

The questionnaires were made available via the Google Forms platform and were scattered through the National Coaches Association, Portuguese Handball Federation, Madeira Handball Association, European Handball Federation, and contacts with researchers from Spain, Iceland, and Uruguay who carried out its dissemination.

#### 2.1.7 Statistics

A descriptive analysis (absolute and relative frequencies) of data from the games was performed via GSEQ software version 5.1.23. The lag sequential analysis was applied following the procedures by Bakeman and Quera (40, 47). Hoisan software version 1.6.3.3.6 was used to analyze the data using the polar coordinate technique (48). Descriptive statistics (mean and standard deviation) were used to characterize the sample collected through the questionnaire, in the variables under study. The dependence between nominal and ordinal variables was determined using the chi-square test, for example between the coaches’ training and the perception and use of 7 vs. 6 EG. Spearman’s correlations were used to determine the association between ordinal variables. The significance level adopted was 5%. The software used was SPSS version 27.0.

## 3 Results

### 3.1 Descriptive analysis from observational data

A descriptive analysis of the data (absolute and relative frequencies) was initially carried out, which allowed decisions to be made regarding subsequent analyses, both lag sequential analysis and polar coordinate analysis, as recommended by Anguera (49) and previously carried out and justified by Prudente et al. (7). A total of 4,236 events were recorded at the 391 offensive sequences registered using 7 vs. 6 EG strategy during the 132 games played by teams ranked 1–12 at four major tournaments between 2020 and 2023 (Euro 2020 and 2022, and WCh 2021 and 2023). The results show that the total number of goals scored in a 7 vs. 6 EG game situation were lower in the WCh compared to those in the Euro, following the trend of reducing the use of this strategy from one tournament to the other. An interesting fact is the verification of a tendency toward a decrease in effectiveness in the use of the 7 vs. 6 EG from the Euro 2020 (51.2%) to the WCh 2023 (48.5%). However, it can be highlighted that the teams classified in the first six places obtained values between 49% (2022) and 51.2% (2021), while there was a decrease in the effectiveness of the teams classified as 7th to 12th, with values that dropped from 50% to 41.7%, improving to 43.8% in the last competition (WCh 2023) (Table 3).

**Table 3:** Goals scored playing 7 vs. 6 with empty goal and % of efficiency.

Competition	Euro 2020	WCh 2021	Euro 2022	WCh 2023
Total Goals scored in 7 vs. 6	63	39	56	33
% of goals by total sequences	51.2	49.4	46.3	48.5
% of goals/total sequences from teams classified from 1st to 6th place	51.6	49	53.1	50
% of goals/total sequences from teams classified from 7th to 12th place	50	50	41.7	43.8

Euro, Men's European Championship; WCh, Men's World Championship.

Comparison between teams classified between 1st and 6th place and teams classified between 7th and 12th place in each competition.

Table 4 shows that there was an evolution regarding the negative consequences of the use of this tactical option. Teams at Euro 2020 had relative success, considering that they conceded a goal after losing the ball in only 12.2% of cases; although the number of responses from the opponent was high, the effectiveness of that response was not high and resulted in a goal only 28.3% of the time. This relationship changed in the following competitions, with a decrease in the number of sequences (more pronounced in the WCh), in the number of responses from the opponent, and in the number of goals conceded by the teams that attacked 7 vs. 6 EG. Response effectiveness registered an increment that went from 28.3% in Euro 2020, to values of 42.9% and 43.5% and even higher than 50% in Euro 2022. This evolution denotes an improvement in teams handling the opponent responses to the use of the 7 vs. 6 EG strategy.

**Table 4:** Number of offensive sequences, in 7 vs. 6 situation, opponent responses and goals conceded.

Competition	Euro 2020	WCh 2021	Euro 2022	WCh 2023
Number of offensives sequences 7 vs. 6	123	79	121	68
Number of opponent responses	53	28	32	23
Goals conceded by teams attacking 7 vs. 6	15	12	17	10
% of goals conceded by total of responses from opponent	28.3	42.9	53.1	43.5
% of goals conceded by total of 7 vs. 6 attacks	12.2	15.2	14.1	14.7

Euro, Men's European Championship; WCh, Men's World Championship.

### 3.2 Descriptive analysis from questionnaire data

#### 3.2.1 Perception and importance of 7 vs. 6 EG

Approximately three out of four coaches agree with the possibility of playing 7 vs. 6 EG (74.4% vs. 25.6%). There was no association between training, experience, and competition level and the coaches’ opinion on the use of 7 vs. 6 EG (*p* > 0.05). Most coaches reported rarely or never using 7 vs. 6 EG, with coaches having less training (39%), less experience (42%), and coaching teams at lower (regional) levels (51.5%) reporting a higher proportion of never using this strategy (0.458 < *r* < 0.231, *p* < 0.05). It turns out that 40.3% of coaches do not support the application of the 7 vs. 6 EG rule and 35.3% are in favor of eliminating the rule. There were no significant differences in the proportion of coaches in favor and not in favor of the 7 vs. 6 EG rule according to training, experience, and level of competition (*p* > 0.05).

The lowest percentage in favor of eliminating the 7 vs. 6 EG rule was found among coaches with less experience (<5 years) (21.7%), while the percentage was higher among those with 5 to 10 years of experience (50%) and those with more than 10 years of experience (46.2%) (χ^2 ^= 10.998, *p* = 0.004).

#### 3.2.2 The use of 7 vs. 6 EG in training and competition

Coaches declared that the 7 vs. 6 EG rule brings little or no advantage (52.6%), regardless of training, experience, or level of competition (*p* > 0.05). Partial score (77.9%) and game time (69%) are mentioned as factors that influence the use of 7 vs. 6 EG. According to the coaches, being behind in the score (78.1%) and the last moments of the game are the best contexts in which to use 7 vs. 6 EG. The use of this strategy should also be conditioned by the tactical-technical quality of the players (94.3%), the team (89.1%), the experience of the players (84.6%), and the characteristics of the opponent (81.4%). The coaches’ opinion is independent of their training, experience, and the level of competition in which they play (*p* > 0.05). Only one in ten coaches (9.7%) agree with the introduction of the 7 vs. 6 EG rule for players aged 15 year or less. There is a consensus among coaches on the age at which the 7 vs. 6 EG rule should be introduced (the majority argue that the rule should only be introduced from U16 (33.1%) and U18 (41.6%), regardless of training, experience, and level of competition (*p* > 0.05). One in three coaches (33.3%) stated that they never use the 7 vs. 6 EG rule in training, 38.7% use it once a week, 22.7% use it 2–3 times a week, and the remaining 5.3% use it at least four times a week. It was found that coaches with a higher level of training (*r* = 0.193, *p* = 0.018), more experience (*r* = 0.54, *p* < 0.0001), and who currently coach at a higher competitive level (international level) (*r* = 0.330, *p* < 0.0001) reported practicing the 7 vs. 6 EG rule more often.

#### 3.2.3 7 vs. 6 EG strategy and opponent's defensive action

The majority of coaches (82.7%) surveyed indicated that the 7 vs. 6 EG rule affects the defensive system, with the 6:0 (42.9%) and 5:1 (36.5%) defensive systems causing the most difficulties in a 7 vs. 6 EG situation. Leading the attack to finish in a certain area is also mentioned as a defensive strategy in 7 vs. 6 EG (92.9%). A large majority of coaches (96.1%) agree that a fast throw-off mast be used as a strategy to fight the use of 7 vs. 6 EG by the opponent. The 7 vs. 6 EG rule affects space management in attacking play, according to most coaches (78.8%), forcing the opposing defense to retreat to 6 m, which is in line with the coaches’ response regarding the defensive system, when they state that 6:0 is what creates the most difficulty over 7 vs. 6 EG. Therefore, the use of one or two pivots is an important strategy in the 7 vs. 6 EG attack and where the role of the central defender (in the case of the game with a pivot) and the pivots are recognized by the coaches. Among the specific positions, coaches highlight the importance of the pivot (31.4%) and the central playmaker (16%) in 7 vs. 6 EG with approximately one in three coaches (31.4%) highlighting the importance of all players. None of the questions were associated with the coaches' training, experience, or level of competition (*p* > 0.05).

### 3.3 Sequential analysis

**Figure 1:**
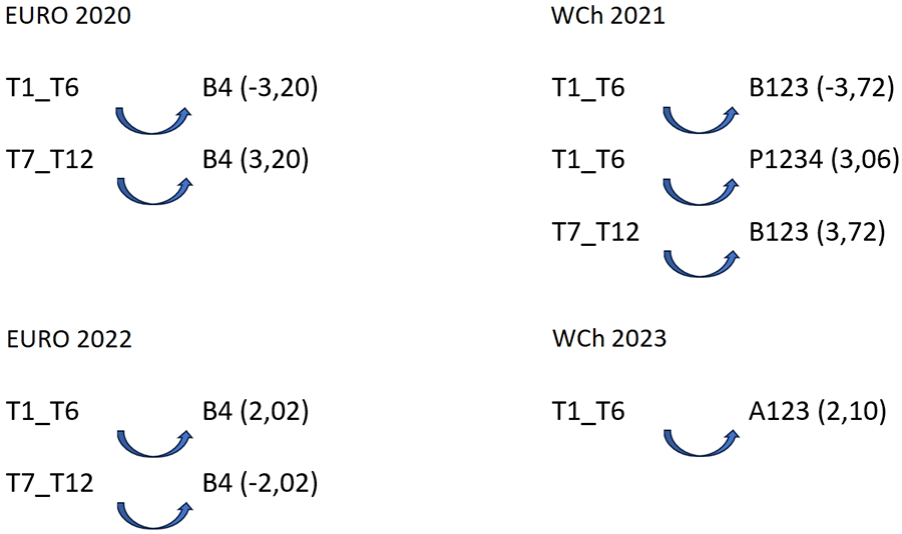
Association pattern between the focal conduct “teams,” considering teams classified between 1st and 6th place and teams classified between 7th and 12th place, and the conditioned conduct “game time”: A123, first half; B123, first 25 min of second half; B4, last 5 min of second half; P1234, extra time period; Euro, Men's European Championship; WCh, Men's World Championship.

A lag sequential analysis (40, 47) was performed to detect some patterns of association (explainable by more than chance) between a given behavior and other variables (50–53). To analyze the probability of association between the focal conduct “teams classified 1st–6th” and “teams classified 7th–12th” and the conditioned conduct “game time” when teams play 7 vs. 6 EG, some patterns were detected (Figure 1).

**Figure 2:**
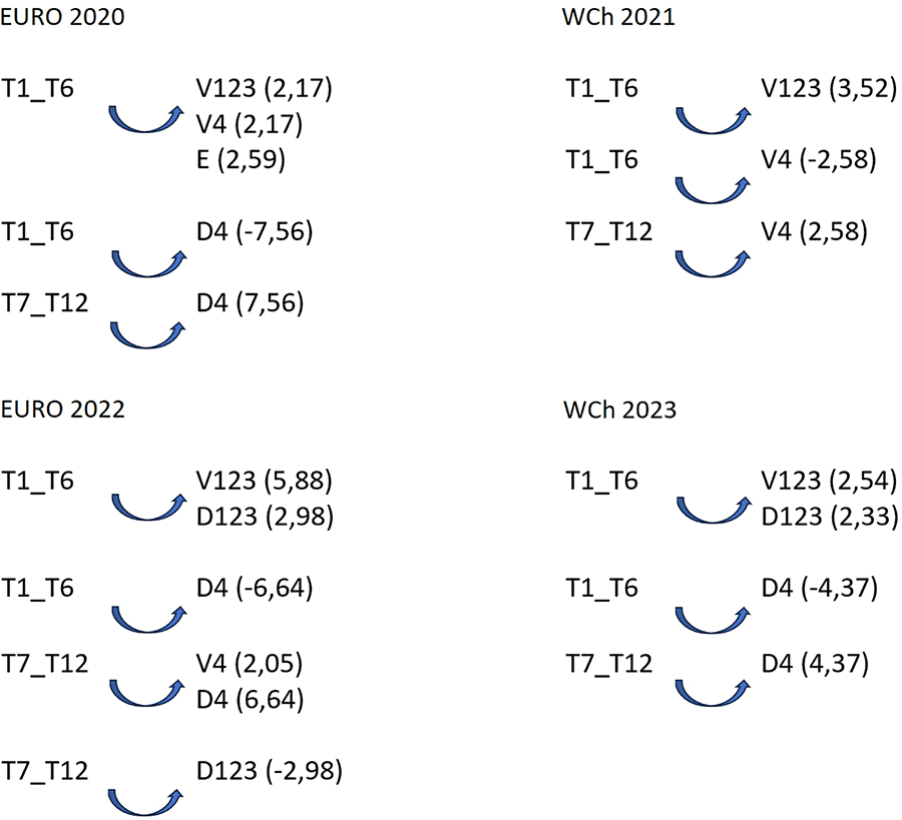
Association pattern between the focal conduct “teams,” considering teams classified between 1st and 6th place and teams classified between 7th and 12th place, and the conditioned conduct “partial score”: V123, winning by 1, 2, or 3 goals; D123, losing by 1, 2, or 3 goals; V4, winning by 4 or more goals; D4 – losing by 4 or more goals; E, draw; Euro, Men's European Championship; WCh, Men's World Championship.

It should be noted that according to the results of the lag sequential analysis, the patterns of regular association, which occur beyond chance, are different in each competition. It should also be highlighted that the teams classified between 7th and 12th place have different patterns compared to the teams ranked among the first six in each competition: the probability of these teams using the 7 vs. 6 EG game in the last 5 min of the second half is significant (B4: 3.20) in Euro 2020, or during the first 25 min of the second half (B123: 3.72) in WCh 2021; however, in Euro 2022, there is a significant probability that teams ranked 7th–12th will inhibit the use of the 7 vs. 6 EG game in the last 5 min of the second half (B4: −2.02), with no pattern of use of 7 vs. 6 EG detected by these teams in WCh 2023. Regarding the teams classified in the top six places, five different patterns were detected: (1) significant probability of inhibition of the use of 7 vs. 6 EG in the last 5 min of the second half (B4: −3.20) in Euro 2020; (2) significant probability of inhibiting the use of 7 vs. 6 EG in the first 25 min of the second half (B123: −3.72); (3) significant probability of using the 7 vs. 6 EG game in extra time (P1234: 3.06) in WCh 2021; (4) significant probability of these teams using the 7 vs. 6 EG game in the last 5 min of the second half (B4: 2.02) in Euro 2022; and (5) using the 7 vs. 6 EG game in the first 25 min of the first half (A123: 2.02) in WCh 2023. To analyze the probability of association between the focal conduct “teams classified 1st–6th” and teams classified “7th–12th” and the conditioned conduct “partial score” (the result on the moment that begins the 7 vs. 6 EG rule), some patterns were detected (Figure 2).

**Figure 3:**
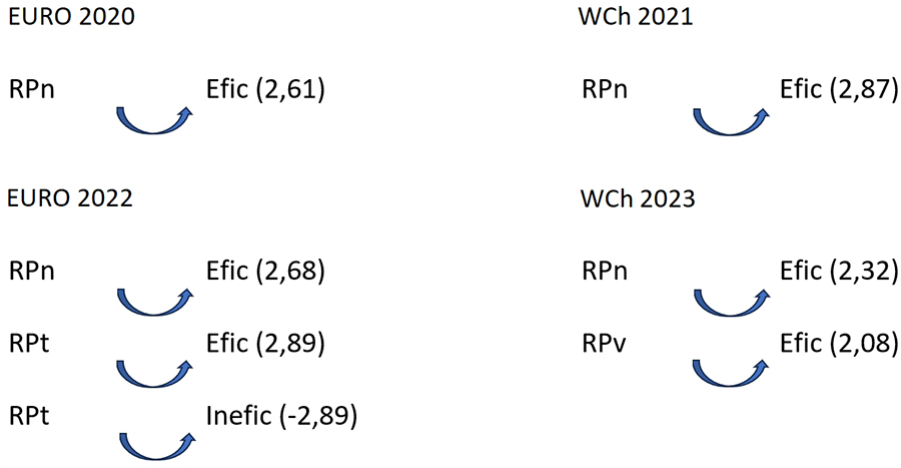
Association pattern between the focal conduct “finalization mode”: breakthrough shot (RPn); wing shot (RPt); pivot shot (RPv), the conditioned conducts “efficiency,” goal or 7 m (Efic), and no goal (Inefic). Euro, Men's European Championship; WCh, Men's World Championship.

In all competitions, some patterns were detected highlighting an association between “partial score” and final ranking. The results showed a difference between teams classified in the first six places and the teams classified as 7th–12th: teams classified in the 7th–12th positions decided to start using the 7 vs. 6 EG rule when they were losing by four or more goals (D4), as confirmed by adjusted residuals for each competition: Euro 2020 (7.56); Euro 2022 (6.64); and WCh 2023 (4.37). However, during WCh 2021, a different pattern was found: they used 7 vs. 6 EG when winning (V4) by four or more goals (2.58). For teams classified in the first six places, losing by four or more goals, was a pattern to inhibit the use of 7 vs. 6 EG (D4): Euro 2020 (−7.56); Euro 2022 (−6.64); and WCh 2023 (−4.37). At WCh 2021, it was found statistically significant that these teams, when they were winning by 1–3 goals (V123), used the tactical option 7 vs. 6 EG (3.52) as it was the case at WCh 2023, when they were losing by 1–3 goals (D123), a pattern of significant association was detected between teams and the D123 (2.98 and 2.33, respectively). Association patterns were found between the focal conduct “finalization mode” (RPn) (breakthrough shot) and the conditioned conducts “efficiency” (Efic) (goal or 7 meters) and “Inefic” (no goal) as shown in Figure 3.

**Figure 4:**
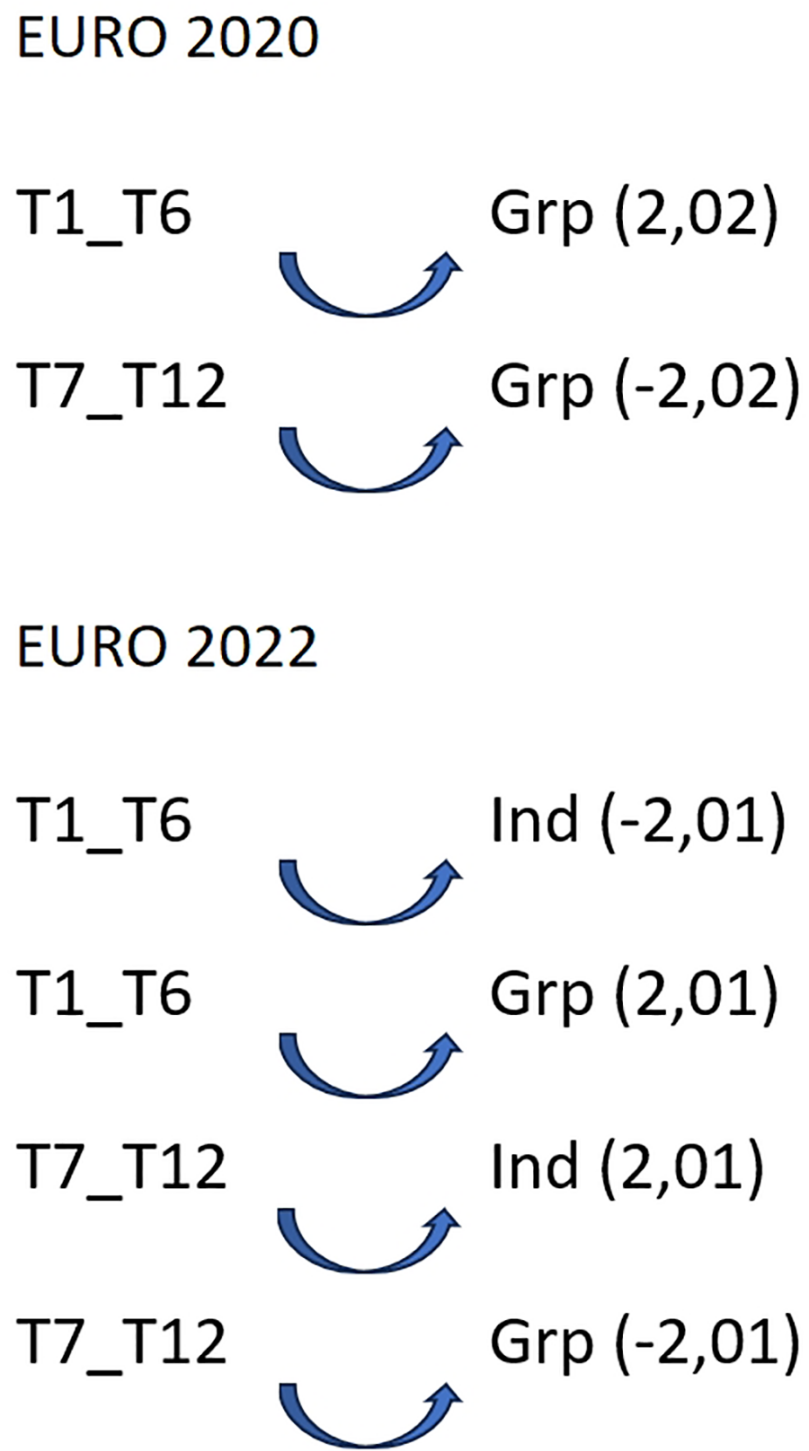
Association pattern between the focal conduct “teams classified 1st–6th” and “teams classified 7th–12th” and the conditioned conduct “tactical means” when teams play 7 vs. 6: Ind, individual tactical means; Grp, group tactical means; Euro, Men's European Championship.

To find a regular association pattern between focal conducts based on teams' ranking (“teams classified 1st–6th” and “teams classified 7th–12th”) and the conditioned conducts based on “tactical means” (“Ind”: individual tactical means and “Grp”: group tactical means), a sequential analysis was performed when the teams played 7 vs. 6 EG (Figure 4).

As observed in Figure 4, in the two European tournaments, teams ranking 1st–6th activated the use of group tactical means (Grp) (Euro 2020 (2.02) and Euro 2022 (2.01)) as well as inhibited the use of individual tactical means in Euro 2022 (−2.01). For teams ranking 7th to 12th, the results showed quite the opposite: inhibiting the use of group tactical means in both Euro 2020 (−2.02) and Euro 2022 (−2.01), and with a significant probability that those teams used individual tactical means (2.01) when playing 7 vs. 6 EG. No patterns were found in WCh 2021 and 2023.

### 3.4 Polar coordinate analysis

A second analysis was performed by applying the polar coordinate technique. While a sequential analysis performs a prospective or retrospective analysis, a polar coordinate analysis performs a prospective and retrospective analysis and gives a vector map that shows how the different variables of the system interact (48). This technique allows us to determine the angles and quadrants in which the vector is located, as well as its length, establishing the type of relationship between the focal conduct (given) and the conditioned conducts defined for each analysis. As mentioned by Prudente et al. (7), the length of the vector expresses the quantitative relationship between the focal conduct (given) and the conditioned conducts (the longer the vector, the stronger the intensity of the relationship between the conducts). The quadrant where the vector is placed expresses the qualitative relationship between these behaviors as follows (6, 54, 55):

Quadrant I (+ +): mutually excitatory given conduct and matching conduct.
Quadrant II (− +): inhibitory given conduct and excitatory matching conduct.
Quadrant III (− −): mutually inhibitory given conduct and matching conduct.
Quadrant IV (+ −): excitatory given conduct and inhibitory matching conduct.

**Figure 5:**
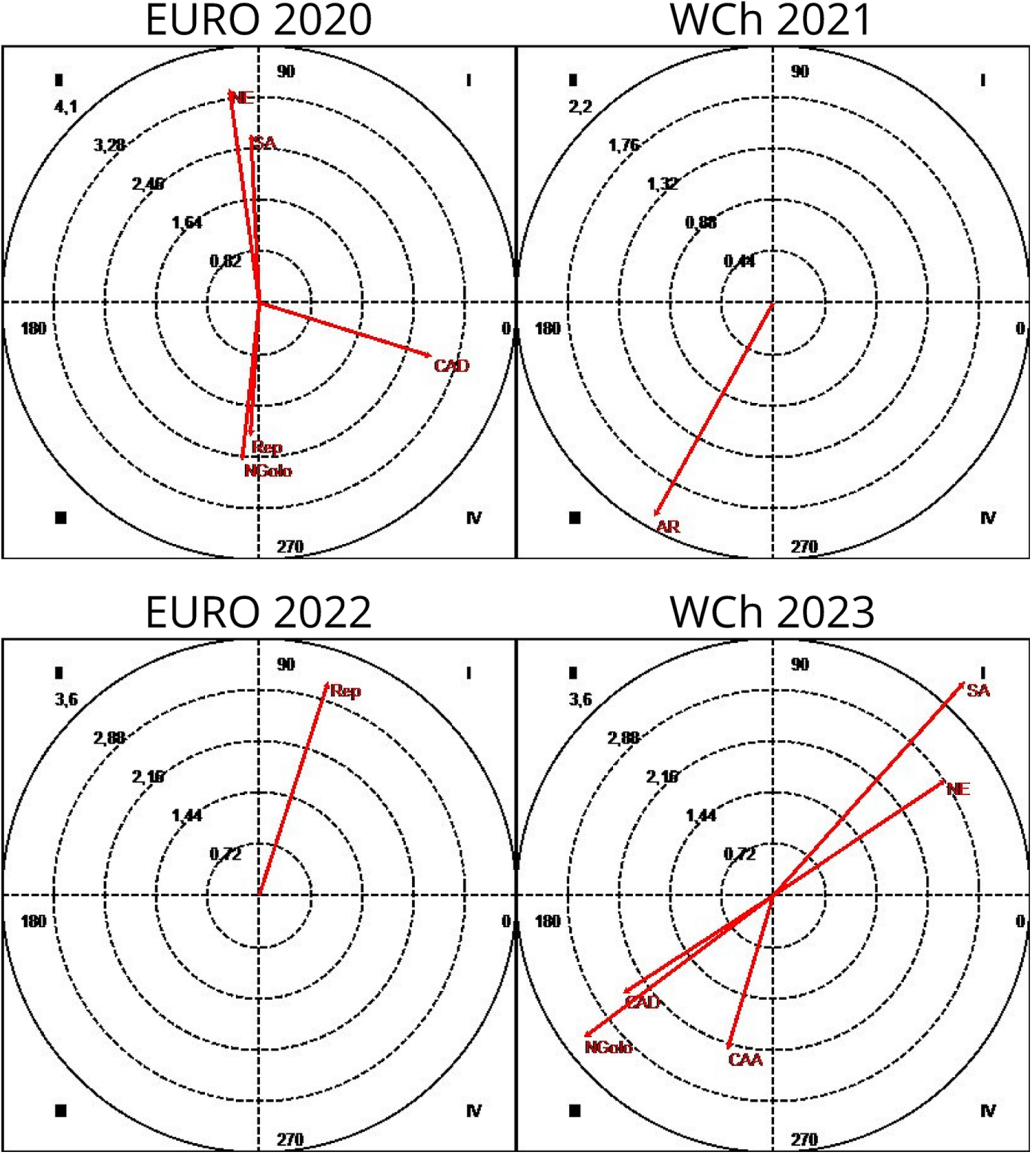
Polar coordinate analysis map: focal conduct “7 m goal” (7MG); conditioned conducts: fast attack (AR); throw-off (Rep); no attack (NE), teams abdicated of fast response; direct fast break (CAD); sustained fast break (CAA); no response (SA); goal, when the opponent response finished scoring a goal (Golo). Euro, Men's European Championship; WCh, Men's World Championship.

Considering the focal conduct “7-m goal” (7MG) and conditioned conducts “throw-off” (Rep), “fast break” (CAD), “fast attack” (AR), “sustained fast break” (CAA), “no attack” (NE), and “no response” (SA), the conduct maps in Figure 5 were obtained. A first analysis of the vector maps shows the differences between the different competitions, including contradictory results. In none of the competitions analyzed did the 7MG conduct trigger the occurrence of an own goal. It is also worth noting that although this focal conduct activated fast REP in Euro 2022 and CAD in Euro 2020, the result of the goal response did not obtain significant results associated with 7MG, as a conduct inhibited or activated. However, it inhibited AR in WCh 2021, and in WCh 2023 it inhibited CAD and CAA, activating NE and SA as a response from the team that conceded the goal from 7 m.

**Figure 6:**
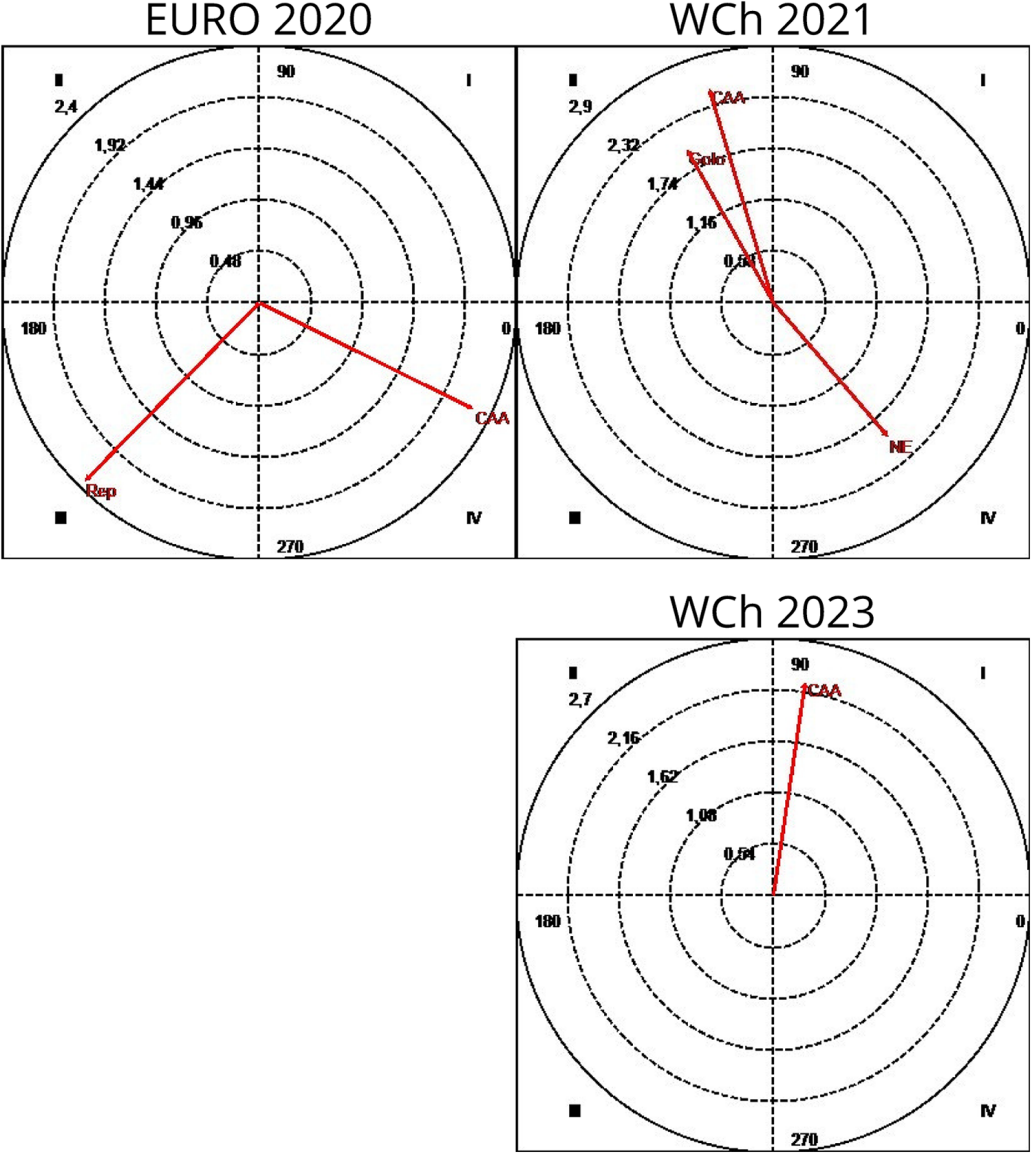
Polar coordinate analysis map: focal conduct “7 m no goal” (7MNG); conditioned conducts: throw-off (Rep); No attack, teams abdicated of fast response (NE); sustained fast break (CAA); goal, when the opponent response finished scoring a goal (Golo). Euro, Men's European Championship; WCh, Men's World Championship.

Figure 6 shows the vector maps that explain the type of relationships found between the focal conduct “7 m no goal” (7MNG) and the conditioned conducts: “no attack” (NE)—teams abdicated of fast response; CAA—sustained fast break; and Golo when the opponent response finished scoring a goal. Some relationships were found between the focal conduct 7MNG and the conditioned conducts: NE, CAA, and Golo. At Euro 2020, the focal conduct 7MNG activated the CAA and there is the existence of a mutually inhibitory relationship with Rep. On the other hand, at WCh 2021, the focal conduct 7MNG inhibits the occurrence of the CAA and is activated by it; and finally, at WCh 2023, the focal conduct 7MNG activates the CAA in a mutually excitatory relationship. At Euro 2022, no significant patterns of relationship between 7MNG and conditioned conducts were detected. However, at WCh 2021, a relation of inhibition of the Golo conduct, relative to the result of the opponent's response and activation of the focal conduct by it, was found as well as the existence of a relationship between the activation of the behavior NE and the inhibition of 7MNG.

**Figure 7:**
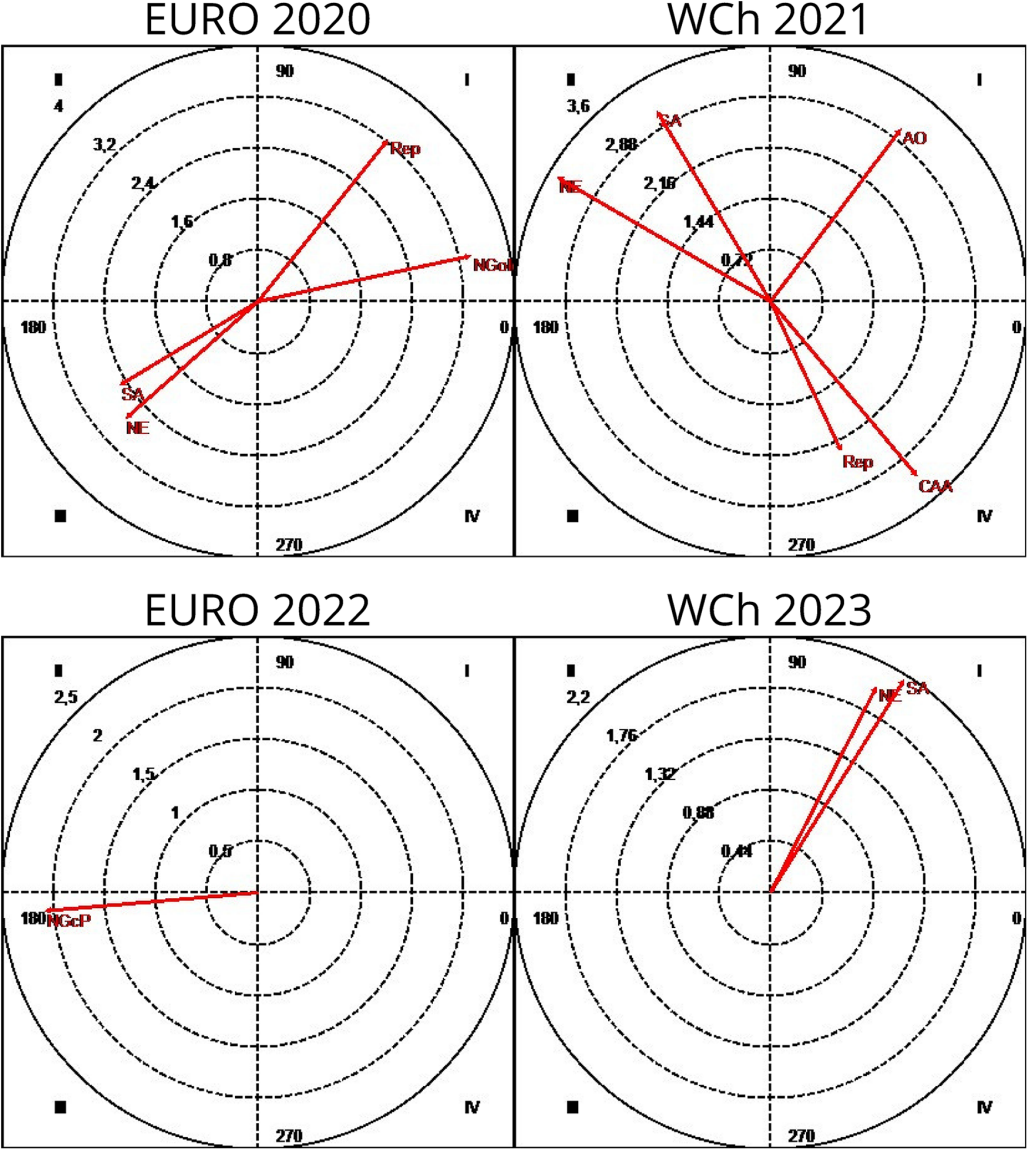
Polar coordinate analysis map: focal conduct “goal” (G); conditioned conducts: throw-off (Rep); no attack, teams abdicated of fast response (NE); sustained fast break (CAA); no goal, when the opponent response finished with a shot but no scoring a goal (NGolo); organized attack (AO); goal, when the opponent response finished with a shot scoring a goal (Golo); no response (SA). Euro, Men's European Championship; WCh, Men's World Championship.

As can be seen, based on the vector maps in Figure 7, obtained considering the focal conduct “G” (goal) and the “opponent's response” and its “opponent response result” as conditioned behaviors, a relationship of mutual activation was detected between G and Rep and Ngolo at Euro 2020, as well as a mutually inhibitory relationship between the same focal conduct and both NE and SA; on the map referring to WCh 2021, we observe the existence of a relationship of mutual activation between the focal conduct G and the AO and a relationship of rapid activation of Rep and CAA being inhibited by these. In Euro 2022, we only detected a significant relationship, of mutual inhibition, between the conduct NGcP and the focal conduct. Finally, at WCh 2023, we found two patterns of mutually excitatory relationship between the focal behavior G and the conditioned behaviors NE and SA.

**Figure 8:**
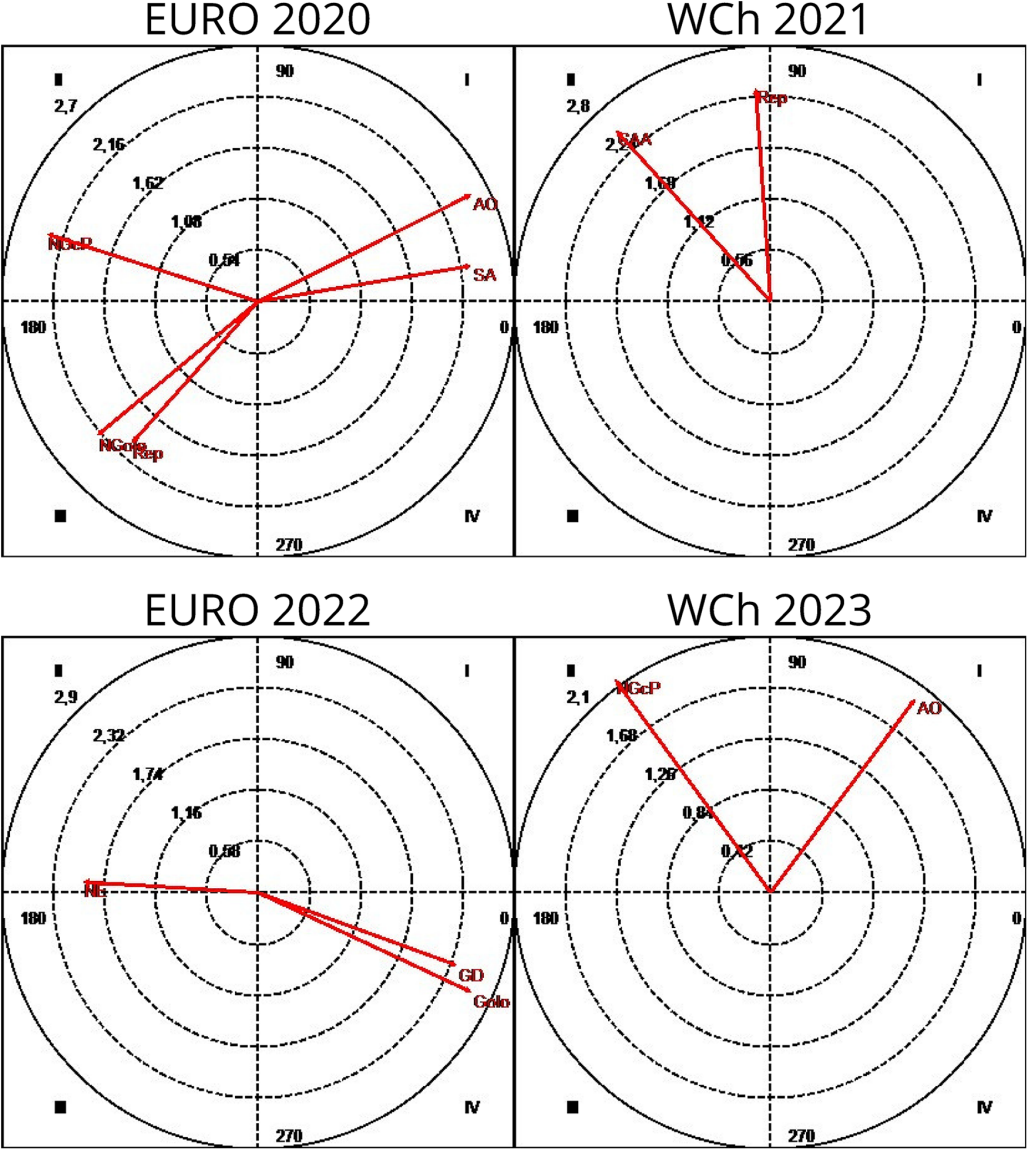
Polar coordinate analysis map: focal conduct “no goal” (NG); conditioned conducts: throw-off (Rep); no attack, teams abdicated of fast response (NE); sustained fast break (CAA); no goal, when the opponent response finished with a shot but no scoring a goal (NGolo); organized attack (AO); no response (SA); goal, when the opponent response finished scoring a goal (Golo); goal-to-goal attempt (GD); no goal with penalty (NGcP). Euro, Men's European Championship; WCh, Men's World Championship.

Figure 8 presents the results having “no goal (NG)” as focal behavior and “opponent's type of response” as conditioned conducts. At Euro 2020, the focal behavior NG presents a mutually excitatory relationship with the conditioned behaviors AO and SA, inhibiting the occurrence of NGcP and being activated by it. NG it also presents a mutually inhibitory relationship with Ngolo as a result from opponent response and Rep behaviors. At WCh 2021, the following significant relationships were detected: NG inhibits and is activated by Rep and CAA. At Euro 2022, an activation relationship was detected between the NG focal conduct and the conditioned conducts GD and Golo when the opponent response finished scoring a goal, which in turn inhibited the NG focal conduct; NG inhibits NE and is activated by it. Finally, at WCh 2023, the focal conduct NG presents a mutually excitatory relationship with the AO conduct and inhibits NGcP being activated by it.

## 4 Discussion

The aim of this study was to contribute to deepening the knowledge of the use of 7 vs. 6 EG in handball, with an approach in which the observation of the teams’ and players’ behaviors during game actions was crossed with the coaches’ perceptions and opinions about this strategy. Therefore, the study tries to understand the evolution of the 7 vs. 6 EG in the last 4 years of men's high-level international competitions (two Euro and two WCh) and verify how the perceptions and opinions of the coaches were whether confirmed or not by the results of the observation.

Based on the results obtained from the opinion of the coaches surveyed, the majority (74.4%) agree with this rule of playing with 7 vs. 6 EG, similar to the coaches’ opinions reflected by Sousa et al. (23), which was 86.3%. Approximately one-quarter of those surveyed in the study (25.6%) was not in agreement, which was higher than the 13.7% found in the study by Sousa et al. (23), pointing out that the controversy generated by this rule has not yet been resolved. Although 64.5% of coaches stated that they did not agree with the elimination of this rule, in line with the 66% found in the study by Sousa et al. (22), 59% supported changes to it, which confirms that the debate continues among coaches. Complementing this opinion, most coaches (55.6%) questioned said they rarely or never used this tactical option, similar to the 56% found by Sousa et al. (23). According to Gilio et al. (29), the coaches highlighted two game situations where the option of attacking with an empty goal arises: when the team is outnumbered and thus seeks to balance and play 6 vs. 6 in attack; and in numerical equality when the aim is to improve the effectiveness of the attack by playing in superiority 7 vs. 6 EG. Moreover, Branco et al. (28) stated that coaches declared to use this strategy to slow down the pace of the game, reduce the physical contact of the players with the opponents, and in moments of the game when their team faces difficulties solving 6 vs. 6 situations in positional attack. Those statement refers to the occasional use of this possibility during the game, thus corroborating the results obtained in the present survey of coaches, where 62.8% of coaches claimed to use 7 vs. 6 EG rarely or sometimes. These statements are sustained by the observational data obtained at men's elite-level handball in the present study (from tournaments played between 2020 and 2023), which showed a decreasing tendency in the number of attacks carried out in 7 vs. 6 EG, going from an average per game of 3.61 7 vs. 6 EG attacks carried out in 2020 to 2.32 in 2021 and 1.89 in 2023 (despite an increase to 4.32 in 2022). The findings are similar to the registered decrease from 6.5% to 4.5% in the use of this strategy in the 2016–2017 and 2017–2018 German Men's First Handball League (19) as well as the decline observed at the Men's Euro and WCh between 2017 and 2020 (56).

The main reason for the low use registered may be related to training aspects, as 33.3% of coaches stated they never train the 7 vs. 6 EG game or only train once a week (38.7%) and only 22.7% train this game situation two or three times a week, which was also confirmed by players’ statements (24) on training once a week (52.6%) or even zero times a week (20%). The trends toward a decrease in the percentage of goals scored in the total of 7 vs. 6 EG sequences carried out by teams in the present study (52.21% in Euro 2020, 49.36% in WCh 2021, 46.28% in Euro 2022, and a slight recovery to 48.53% in WCh 2023) could also be interpreted as a stabilization on the efficiency in attacking 7 vs. 6 EG. These results are also similar to the decrease in the novelty and efficacy in the use of the 7 vs. 6 EG strategy registered at men's Euro and WCh from 2017 to 2020 (56).

The results also show that the percentage of goals conceded, by the teams that attacked 7 vs. 6 EG in the opponent's total responses when they recovered the ball, went from 28.3% in Euro 2020 to 42.85% in WCh 2021 and 53.13% at Euro 2022, and then dropping to 43.48% in WCh 2023. The evolutionary trend in the percentage of goals scored when using 7 vs. 6 EG (in the range of 46.28%–52.21%), associated with an increasing trend in the percentage of goals conceded in relation to the responses of the teams that were defending against the 7 vs. 6 EG attack (range 28.3%–53.13%), leads to the interpretation that the teams went from a less structured and trained phase of using 7 vs. 6 EG to a phase of adaptation to this new rule. This was confirmed when 44.2% of the coaches surveyed agreed that teams using this tactical option must first carry out specific work. As can be seen from the results obtained from games data, teams were using this tactical option with more security. This indicates that tactical and strategic solutions were found in order to reduce the successful use of the 7 vs. 6 EG option. When teams attack 7 vs. 6 EG, it becomes necessary to successfully finish the attack scoring, so as not to allow the opponent to take advantage of the transition between the departure of the additional field player and the re-entry of the goalkeeper, as noted by Gilio et al. (29). This adaptation was also caused by an increase in the speed of play, because of the ball being placed quickly in the center of the court followed by a fast throw-off being performed. The fact is that reducing the time that the opposing goalkeeper has to return to the goal when replacing the additional field player will thus dissuade the opponent from using the option of playing 7 vs. 6 EG. Through this behavior of fast “throw-off,” a quick response from the team defending 7 vs. 6 EG when conceding a goal can be performed. However, it should be noted that if we consider the percentage of goals conceded by the teams that opted for the 7 vs. 6 EG attack in relation to the total number of sequences in 7 vs. 6 EG, the variation was smaller: 12.20% at Euro 2020 and a maximum of 15.19% at WCh 2021, having stabilized in the last two competitions analyzed at 14.05% (Euro 2022) and 14.71% (WCh 2023).

The results obtained with the polar coordinated analysis, when considering the focal conduct “goal” (G) and conditional conducts the response of the other team, patterns of associations of mutual activation in the different championships were observed, but no significant results of activation relationship was found between the focal conduct “goal” (G) and the “goal” conduct (Golo) resulting from the defending team's response action. That means no significant negative consequences were found for teams that decide to use 7 vs. 6 EG option when they scored a goal. When performing the same analysis to identify some relation between the focal conduct “no goal” (NG) and the conducts related to the responses of the other team, a significant relation was found only at Euro 2022 when “no goal” (NG) activate “goal” (Golo) was found, but this one inhibits “no goal” (NG). All these results are in line with the findings cited by Gümüş and Gencoglu (17), highlighting that teams using 7 vs. 6 EG did not have greater efficiency in attack, nor did the use of this tactical option have negative consequences or increase the risk of conceding a goal for these teams. Moreover, the results are similar to those found by Trejo-Silva and Bonjour (56), which stated a better successful finalization efficacy when playing 6 vs. 6 with the goalkeeper at the goal (48.9%) against 7 vs. 6 EG (41.9%).

The coaches surveyed have the perception that 7 vs. 6 EG brings little or some advantage (52.6%). However, they indicate that “partial score” (77.9%) and “game time” (69%) influence whether 7 vs. 6 EG is used. Coaches refer to being behind in the score (65.8.1%) and the final moments of the game as privileged situations in which to use 7 vs. 6 EG, while 9.6% of coaches refer to using it when they are tied and only 5.3% when they are winning. Prudente et al. (27) obtained similar results, since most of coaches (65.8%) consider that game time influences the use of the 7 vs. 6 strategy with an “empty goal,” 92.2% consider that this use occurs in the final moments of the game, and 74.7% of respondents stated that “partial score” influences the use of 7 vs. 6 EG, with 90.7% considering that the team being at a disadvantage also influences its use. Haugen and Guvåg (15) mentioned that an additional field player will allow numerical superiority in attack, both for the best and the worst teams; however, according to these authors, the risk of playing with an empty goal can allow a more skilled opponent to win the ball, both for goalkeeper saves as per a better defensive behavior and scoring easily in an empty goal.

The results of the lag sequential analysis carried out to analyze the probability of association between the focal conducts “teams classified 1st–6th” and “teams classified 7th–12th” and the conditional conduct “game time” when teams play 7 vs. 6 EG has detected some patterns that confirm the opinions and perceptions of the coaches: “game time” activates the use of 7 vs. 6 EG by teams. Regular association patterns of using 7 vs. 6 EG. were detected during the following periods of game time: between 30′ 01″ and 55′ of the second half (“B123”, 3.72); the period of the game between 55′ 01″ and 60′ of the second half (“B4”, 2.02), the extra time period of the game (“P1234”, 3.06); and, only at WCh 2023, between 0′ and 25′ of the first half (“A123”, 2.10). Therefore, it can be confirmed that the probability of the game periods in the second half and extra time (“B123,” “B4,” and “P1234”) being associated with the use of 7 vs. 6 EG is significant. This confirms the results of Gilio et al. (29), who stated that the use of the empty goal was related to the characteristics of the available players and the context of the game, particularly the game score and time. In addition, the results by Neuberg and Thiem (19) showed that the efficiency of using an additional field player depends, in a way, on timing, as was also declared by coaches when interviewed by Branco et al. (28) and Krahenbühl et al. (25, 26). The coaches’ perception that “partial score,” namely, when losing, is a prime situation in which to start using 7 vs. 6 EG, as expressed by 78.1% of questioned coaches, is confirmed by the results from the sequential analysis that show the teams classified 7th–12th when losing by four goals (D4) presented a significant probability to use this strategy at Euro 2020, Euro 2022, and WCh 2023. For teams classified between 1st and 6th places, a pattern of regular association with partial score when losing by one, two, or three goals (D123) was found at Euro 2022 and at WCh 2023. These results are in line with those in the study by Prudente et al. (7), where the teams show they preferred using 7 vs. 6 EG when the result was a momentary defeat by one, two, or three goals (D123) and they were behind by four or more goals (D4).

According to Gilio et al. (29), in the study carried out on the opinion of Brazilian coaches regarding the use of the additional field player, all coaches highlighted that the result associated with playing time influences whether the additional field player will be used. The authors also stated that game time proved to be relevant in the decision to opt for the 7 vs. 6 EG attack, especially when the teams were losing in the final minutes of each half of the game. Moreover, coaches also stated that playing 7 vs. 6 EG prevents defensive pressure, and a slightly more open defense, forcing the opponent to play more with blocking and less with interception, pressure, and direct contact, suggesting that short movements to attack empty spaces were recommended, to the detriment of one-on-one actions. Moreover, coach participants in the study by Branco et al. (28) also stated the same idea when analyzing the context of using 7 vs. 6 EG in terms of controlling the physical impact during a match or even during a period of a tournament. The results of the lag sequential analysis performed in this study apparently confirm this assumption: the results obtained show the existence of regular patterns of association.

Regarding the type of shot, the results show that the “penetration shot” (RPn) is associated with the effectiveness of the attack in all observed competitions and the “pivot shot” (RPv) at the WCh 2021.

When analyzing the use of the tactical means by teams, although we only achieved significant results at Euro 2020 and Euro 2022, we found differences between teams classified from 1st to 6th to the teams classified between 7th and 12th place, where the better classified teams activate group tactical means and the other teams activate the individual tactical means. In the analysis performed with the lag sequential analysis and the polar coordinate analysis, we did not find any pattern where the 9 m shot was a part of a pattern detected. Prudente et al. (7) also analyses the type of shot and observed that the 6 m shot was most common in all teams observed when they play 7 vs. 6 EG, considering it is a consequence of most teams choosing to play with two wing players and two pivots.

## 5 Conclusion

The results obtained and analyzed allow us to state that coaches, despite agreeing that the 7 vs. 6 EG rule corresponds to an evolution of the game, defend changes to it. Considering the results of the survey and the analysis of the observational records, it can be seen that the use of the rule by teams has evolved, with more punctual use and ensuring countermeasures to avoid conceding a goal in the opponent's response. Teams use this strategy mainly when they are at a disadvantage on the scoreboard, highlighting a difference between the teams ranked 1st–6th and the teams ranked 7th–12th: the former use the 7 vs. 6 EG game in a more varied way in “game time” and “partial score,” while the latter opt for the 7 vs. 6 EG rule when they are behind by four goals or more and especially at the end of the game. The results indicate that training in this specific game situation still does not occur frequently to improve the efficiency of the 7 vs. 6 EG attacks. Clearly, this is an important indication for coaches.

## 6 Future research

Future research should also consider field players and goalkeepers, in addition to coaches, as well as data obtained through the systematic observation of player and team behavior. It will also be important to consider the 6 vs. 6 EG game in addition to 7 vs. 6, and use the T-pattern in data analysis, to discover and analyze hidden repeated temporal and often multimodal patterns in behavior, as mentioned by Pic et al. (38).

## Data Availability

The raw data supporting the conclusions of this article will be made available by the authors, without undue reservation.
