# Supplementary material for: Evolution of attack in handball when playing 7 vs. 6 with empty goal between 2020 and 2023: coaches’ perception vs. observational results
Source: Front Sports Act Living. 2024 Mar 14;6:1354623. doi: 10.3389/fspor.2024.1354623 (PMC10973547; doi:10.3389/fspor.2024.1354623)
Supplement: Supplementary file 1 [file Datasheet1.pdf]

Observation instrument

Criteria	Description	Label	Codes	Label	Code
Teams	*Teams according to their order at the competition final table*	1st position<br>2nd position<br>3rd position<br>4th position<br>5th position<br>6th position	T1<br>T2<br>T3<br>T4<br>T5<br>T6	7th position<br>8th position<br>9th position<br>10th position<br>11th position<br>12th position	T7<br>T8<br>T9<br>T10<br>T11<br>T12
Game time	*Periods of game time*	0´-10´minuts of play<br>10´01´´- 20´<br>20´01´´- 30´<br>30´01´´- 40´<br>40´01´´- 50´<br>50´01 - 55´	A1<br>A2<br>A3<br>B1<br>B2<br>B3	55’01’’- 60’<br>60’’01’’-65’<br>65’01’’ – 70’<br>70’01’’ – 75’<br>75’01’’ – 80’	B4<br>P1<br>P2<br>P3<br>P4
The partial score	*The result on the moment that begin the 7 vs. 6 situation*	Draw<br>Victory by one<br>Victory by two<br>Victory by three<br>Victory by four or more	E<br>V1<br>V2<br>V3<br>V4	Defeat by one<br>Defeat by two<br>Defeat by three<br>Defeat by four or more	D1<br>D2<br>D3<br>D4
Defensive system	*Organized defence*	Defence Zone 6:0<br>Defence Zone 5:1<br>Defence Zone 3:2:1<br>Defence Zone 3:3	6_0<br>5_1<br>3_2_1<br>3_3	Defence Zone 4:2<br>Defence Mixte 5+1<br>Defence Mixte 4+2<br>Individual defence	4_2<br>5+1<br>4+2<br>H_H
Offensive organization	*Organized attack related with number of pivot players*	Attack with one pivot player	1Pv	Attack with two pivots players	2Pv
Tactical means	*Tactical means used during attack 7vs6*	Individual tactic means	Ind	Group tactical means	Grp
Shot	*Kind of shot used to score*	9 meters shot<br>Wing shot<br>Pivot shot	R1L<br>RPt<br>RPv	Breakthrough shot<br>No shot	RPn<br>SR
Attack Result	*Result after shot to the goal*	Goal<br>No Goal<br>7 Meters with Goal	G<br>NG<br>7MG	7 Meters No Goal<br>No Shot by Technical Fault<br>No Shot by Opponent action	7MNG<br>SFFt<br>SFAa
Zone	*The shot zone of the offensive sequence*	Z1 – Left wing attack<br>Z2 – 6 mts – line player<br>Z3 – Right Wing attack<br>Z4 – Left back from 9 to 15m<br>Z5 – Centre Back from 9 to 15m	Z1<br>Z2<br>Z3<br>Z4<br>Z5	Z6 – Right back from 9 to 15m<br>Z7 – Left From 15 to 20m<br>Z8 – Centre From 15 to 20m<br>Z9 – Right from 15 to 20m<br>Z10 – Defensive area including GK area	Z6<br>Z7<br>Z8<br>Z9<br>Z10

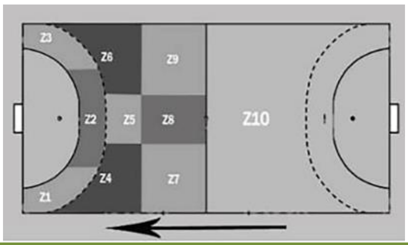

Opponent Response	*Opposing team´s response after regaining possession of the ball*	Goal-to-Goal Attempt<br>Direc6t Fast Break<br>Sustained Fast Break<br>Throw-off	GD<br>CAD<br>CAA<br>Rep	Fast attack<br>Organized Attack<br>No Response	AR<br>AO<br>NE
Opponent Response Result	*Result of the opposing team´s response*	Goal when the opponent response finished scoring a goal<br>No Goal when the opponent response finished with a shot but no scoring a goal	Golo<br>NGolo	No Goal with Penalty<br>No Goal with Opponent Action	NGcP<br>SA
